# Approaches to management of cardiovascular morbidity in adult cancer patients – cross-sectional survey among cardio-oncology experts

**DOI:** 10.1186/s40959-020-00070-y

**Published:** 2020-09-01

**Authors:** E. Hedayati, A. Papakonstantinou, A. Månsson-Broberg, J. Bergh, L. Hubbert, R. Altena

**Affiliations:** 1 Department of Oncology and Pathology Cancer Center Karolinska, Karolinska Institutet, Stockholm, Sweden; 2 Medical unit breast, endocrine tumors and sarcoma, Theme Cancer, Karolinska University Hospital Stockholm, Solna, Sweden; 3 Cardiology Unit, Theme Heart, Vascular and Neuro, Karolinska University Hospital Stockholm, Solna, Sweden; 4 Department of Medicine Huddinge, Karolinska Institutet, Huddinge, Sweden; 5 Department of Cardiology and Department of Health, Medicine and Caring Sciences, Linköping University, Norrköping, Sweden; 6 Transplant Institute, Sahlgrenska University Hospital, Gothenburg, Sweden

**Keywords:** Cardio-oncology, Management, Prevention, Expert-based opinion

## Abstract

**Background:**

In cardio-oncology, a range of clinical dilemmas can be identified where high-quality evidence for management is still lacking. The aim of this project was to study clinical practices and expert approaches to several clinical cardio-oncological dilemmas regarding prediction, prevention and treatment of cardiovascular disease in adult cancer patients.

**Methods:**

A cross-sectional online survey was sent out to internationally renowned experts in the field of cardio-oncology. Participants were selected based on being first or last authors of papers in the field of cardio-oncology, or principal investigators to trials in this field.

**Results:**

Topics discussed include, among others, the use of biomarkers for subclinical cardiovascular toxicity, approaches towards primary prevention and follow-up with medication and life-style recommendations, and management of fluoropyrimidine-vasospasm, QTc-prolongation and asymptomatic declines in left ventricular ejection fraction.

**Conclusion:**

The answers provided in this survey have shed light on expert-based practices in cardio-oncologic dilemmas. Attitudes towards, as well as discrepancies in those dilemmas are presented. Existing discrepancies clearly indicate the need for generation of high-quality data that allows for more evidence-based recommendations in the future.

## Background

Globally, the incidence as well as survival rates of cancer are rising [1–4]. Many patients diagnosed with malignancies receive multimodality treatment, especially those with an early stage cancer that is amenable to curative treatment. These treatments can consist of different combinations of surgery, radio-, chemo- and targeted therapies. Different treatment modalities have diverse modes of action and range of acute and late side effects. Although the development of treatment-related toxicity has been historically regarded as ‘collateral damage’ of an oncological treatment, efforts are now focusing to prevent and manage toxicity in order to minimize the negative short and long-term effects. This is important for several reasons; reducing acute toxicity may increase the possibility to provide optimally dosed anti-cancer treatment, which in turn improves oncological outcomes. In addition, decreasing late, or chronic, treatment-related toxicity is vital to maximize healthy survivorship and improve quality of life in cancer survivors [5].

The cardiovascular system is at particular risk for cancer-treatment related complications, both on the short and long term. Over the past decades, a global cardio-oncology effort fed by a large society of clinicians and researchers has focused on understanding and ameliorating cardiovascular effects that can occur in cancer patients. Mechanisms of toxicity have been unraveled, and newer projects focus on cardiovascular toxicity from contemporary oncological treatments, such as checkpoint inhibitors. Several intervention studies on primary prevention have been performed [6–11], and some predictive models have been composed to identify cancer patients at increased risk for treatment-related cardiovascular disease (CVD) [12–14].

Despite those advances, clinical dilemmas can be identified and differences in clinical practice exist. The mostly used guidelines on monitoring risk and cardiac dysfunction from the American Society of Clinical Oncology, Canadian Cardiovascular Society [15] and European Society of Cardiology [16] provide expert-based summaries of the relevant literature on risk assessment, prevention and early detection up to 2016. However, due to lack of evidence, they do not cover many of the specific clinical situations that physicians treating patients with toxic cardiomyopathy due to anti-neoplastic treatment encounter, and lack recommendations on which patients should, for example, receive primary prevention by means of prophylactic use of beta-blockers, angiotensin converting enzyme inhibitors (ACEi) and more [17]. The very recently published guideline from the European Society of Medical Oncology provides an excellent overview on a number of clinical dilemmas and concisely summarizes the available literature on these topics, while acknowledging the paucity of conclusive data to formulate firm management recommendations [18].

Hereby, we report findings from a cross-sectional online survey that was performed among cardio-oncology experts. The aim was to study clinical practices and expert approaches to several clinical cardio-oncological dilemmas regarding prediction, prevention and treatment of CVD in adult cancer patients, where evidence for definitive management recommendations is as of yet scarce.

## Methods

### Composition of questions in cross-sectional online questionnaire

The cardio-oncology team at the Karolinska University Hospital is composed of clinically active oncologists (EH, AP, RA) and cardiologists (AMB, LH [prev]), and has scheduled meetings at regular intervals to discuss patients with active oncological and cardiovascular conditions. The survey-questions included in the online questionnaire were composed by the cardio-oncology team based on clinical dilemma’s that arise from the multidisciplinary discussions and were approved by all team members.

### Selection of candidates for online survey

Suitable candidates for the cross-sectional online questionnaire were selected based on a literature review on PubMed. Search words for selection of the articles included ‘cardiotoxicity’, ‘oncology’, ‘cardiovascular toxicity’, ‘cancer’, ‘cardiooncology’, ‘cardio-oncology’. Papers written in English from January 2000 up to September 2018 were included. The first and last authors were added to the list of candidates, if their email address could be retrieved from the corresponding author list, personal connections or an Internet search. In case these contact details could not be retrieved, the corresponding author of the article was instead added to the candidate list.

In addition, the website clinicaltrials.gov was searched for studies related to cancer therapies and cardiotoxicity/cardiovascular toxicity, including studies on imaging, biomarkers, systemic therapy, radiotherapy and interventions. The Principal Investigators of these trials were added to the candidate list.

### Online questionnaire

The questionnaire consisted of 21 multiple-choice questions and one open question requiring a written response. All questions and possible multiple-choice answers are presented in Table 1. For each question, only one of the answers could be selected. As an alternative to the provided answers for every multiple-choice question, there was a possibility to select ‘other’ and write free text instead .

**Table 1:** Questions in online survey. For each question, only one of the answers could be selected. As an alternative, there was the possibility to write free text instead of choosing one of the available options for every multiple-choice question. In the last column, the percentage response to the respective options is given. Additional file 1 includes the remarks that were given at in the open text field at the option ‘other’

Topic	Question	Possible answers	Answers provided in the total group
Demographics	In what professional way are you affiliated to cardio-oncology?	a. Oncologist (medical/clinical/radiation therapy b. Cardiologist c. Pre-clinical researcher d. Epidemiologist e. Other, …	a. 26 (28.0%) b. 48 (51.6%) c. 8 (8.6%) d. 2 (2.2%) e. 9 (9.7%)
	In what kind of institution is your main position?	a. University/teaching hospital b. Community hospital c. Private hospital d. Research institute e. Other, …	a. 83 (90.2%) b. 3 (3.3%) c. 2 (2.2%) d. 2 (2.2%) e. 2 (2.2%)
	How long have you been practicing your profession?	a. > 20 years b. 10–20 years c. 5–10 years d. < 5 years e. In training	a. 38 (41.3%) b. 28 (30.4%) c. 15 (16.3%) d. 7 (7.6%) e. 4 (4.3%)
	How long have you been involved in cardio-oncologic care/research?	a. > 20 years b. 10–20 years c. 5–10 years d. < 5 years e. In training	a. 9 (9.8%) b. 23 (25.0%) c. 28 (30.4%) d. 29 (31.5%) e. 2 (2.2%)
	What is your gender	a. Female b. Male	a. 34 (37.8%) b. 56 (62.2%)
Organization of cardio-oncologic care	How is the care for patients with an oncological diagnosis and cardiovascular disease (CVD) organized at your institution?	a. Dedicated clinical team with scheduled multi-disciplinary conferences b. Ad-hoc multi-disciplinary discussions and referrals c. No possibilities for this d. Other, … e. Not applicable	a. 38 (41.3%) b. 42 (45.7%) c. 5 (5.4%) d. 4 (4.3%) e. 3 (3.3%)
Prevention	Do you prescribe preventive medication before start of a potentially cardiotoxic oncological treatment in patients with a normal left ventricular ejection fraction (LVEF) and without uncontrolled risk factors for cardiovascular disease (CVD)?	a. No b. Yes, an ACE-inhibitor or angiotensin receptor antagonist c. Yes, a beta-blocker d. Yes, a statin e. Yes, anticoagulants f. Yes, a combination of the abovementioned g. Only in case of estimated increased risk for cardiotoxicity based on published risk scores (e.g., Ezaz et al., J Am Heart Assoc 2014; Herrmann et al., Mayo Clin Proc 2014) h. Other, … i. Not applicable	a. 48 (52.7%) b. 4 (4.4%) c. 1 (1.1%) d. 0 e. 0 f. 8 (8.8%) g. 15 (16.5%) h. 6 (6.6%) i. 9 (9.9%)
	Do you provide life-style advices to your patients (with and without CVD) before they commence an oncological treatment?	a. No, no time for this b. No, not enough evidence to support such recommendations c. Yes, physical exercise d. Yes, weight loss e. Yes, smoking cessation f. Yes, a combination of the abovementioned g. Other, … h. Not applicable	a. 4 (4.4%) b. 5 (5.5%) c. 5 (5.5%) d. 0 e. 0 f. 65 (71.4%) g. 5 (5.5%) h. 7 (7.7%)
	What do you regard the best method for prevention of future development of CVD in patients treated for a malignancy?	a. Cardiovascular risk management according to general guidelines, with treatment initiation based on accepted thresholds (blood pressure, lipid profile, glucose) b. Preventive medication such as ACE-inhibitors, ARBs, beta-blockade independent of risk factors c. Preventive medication such as ACE-inhibitors, ARBs, beta-blockade in patients with rises in troponin/NT-proBNP d. Life style management (e.g., physical exercise, weight loss) e. Other, … f. Not applicable	a. 48 (53.3%) b. 6 (6.7%) c. 16 (17.8%) d. 8 (8.9%) e. 5 (5.6%) f. 7 (7.8%)
Follow-up	Is there a standardized follow-up schedule that you use for patients treated with potentially cardiotoxic cancer treatments?	a. Yes, according to the ASCO guideline (Prevention and Monitoring of Cardiac Dysfunction in Survivors of Adult Cancers, 2016) b. Yes, according to childhood cancer survivorship guidelines c. Yes, we have our own local/regional guideline d. No, pragmatic and/or individualized schemes e. Other, … f. Not applicable	a. 28 (31.1%) b. 4 (4.4%) c. 24 (26.7%) d. 20 (22.2%) e. 2 (2.2%) f. (13.3%)
	Who do you deem responsible for CVD risk management in cancer survivors	a. Cardio-oncology outpatient clinic b. Cardiologist c. Oncologist d. General practitioner e. The patient self f. Other, … g. Not applicable	a. 44 (48.9%) b. 12 (13.3%) c. 6 (6.7%) d. 15 (16.7%) e. 1 (1.1%) f. 7 (7.8%) g. 5 (5.6%)
Management – oncological treatment decisions	What is your approach regarding choice of (neo-)adjuvant systemic breast cancer treatment in a patient with an indication for chemotherapy and a previous cardiovascular event, presuming the symptomatology is controlled and the patient has a normal LVEF?	a. Anthracycline- and taxane-based chemotherapy b. Non-anthracycline containing chemotherapy c. No chemotherapy because of co-morbidity d. Other, … e. Not applicable	a. 35 (38.9%) b. 24 (26.7%) c. 0 d. 7 (7.8%) e. 24 (26.7%)
	Do you continue treatment with trastuzumab (in curative and/or palliative setting) in case of an asymptomatic left ventricular ejection fraction (LVEF) drop to < 50% but ≥45%?	a. Never b. Yes, often c. Occasionally, after consultation with cardiologist when adequate anti-congestive treatment is initiated d. I do not routinely check LVEF during trastuzumab treatment if patients do not have any clinical signs of heart failure e. Other, … f. Not applicable	a. 5 (5.6%) b. 33 (36.7%) c. 30 (33.3%) d. 0 e. 5 (5.6%) f. 17 (18.9%)
	How do you respond when a patient develops a prolonged QTc under systemic oncological treatment?	a. Interrupt anti-cancer treatment, follow-up QTc and re-initiate only after QTc has normalized b. Continue treatment with continued monitoring of QTc c. I do not routinely check QTc under anti-cancer treatment d. Other, … e. Not applicable	a. 22 (24.7%) b. 33 (37.1%) c. 14 (15.7%) d. 4 (4.5%) e. 16 (18.0%)
	Do you continue treatment with fluoropyrimidines (5-FU, capecitabin) if a patient has developed an acute coronary syndrome?	a. No b. Yes, after initiation of a calcium-antagonist c. Yes, only if there was no enzymatic myocardial infarction and if anti-angina treatment is started d. Other, … e. Not applicable	a. 28 (31.1%) b. 10 (11.1%) c. 20 (22.2%) d. 10 (11.1%) e. 22 (24.4%)
Management – cardiovascular treatment decisions	What systolic blood pressure do you aim for under treatment with anti-Vascular Endothelial Growth Factor therapy (bevacizumab, tyrosine kinase inhibitors)?	a. < 160 mmHg b. < 140 mmHg c. < 120 mmHg d. Only if there are clinical symptoms of hypertension and/or proteinuria e. Other, … f. Not applicable	a. 6. 6.7%) b. 56 (62.2%) c. 6 (6.7%) d. 1 (1.1%) e. 0 f. 21 (23.3%)
	Do you prescribe novel oral anticoagulants to patients with an oncological treatment?	a. No, because of bleeding risk b. No, because of possible interactions with oncological treatment c. Yes, for atrial fibrillation d. Yes, for venous thrombo-embolism e. Yes, both for atrial fibrillation and venous thrombo-embolism f. Other, … g. Not applicable	a. 4 (4.4%) b. 6 (6.7%) c. 11 (12.2%) d. 2 (2.2%) e. 41 (45.6%) f. 9 (10.0%) g 17 (18.9%)
	Do you, apart from LVEF, use a biomarker for subclinical cardiovascular toxicity for clinical decision making?	a. No b. Yes, circulating biomarkers (NT-proBNP, troponins) c. Yes, global strain on echocardiography d. Yes, diastolic function on echocardiography (E/A-ratio, tissue velocities) e. Yes, cardiac MRI f. Yes, coronary artery calcification scores on computed tomography g. Other, … h. Not applicable	a. 24 (26.7%) b. 23 (25.6%) c. 16 (17.8%) d. 2 (2.2%) e. 2 (1.1%) f. 0 g. 15 (16.7%) h. 9 (10.0%)
	Do you consider placement of an implantable cardioverter-defibrillator (ICD) and/or cardiac resynchronization therapy (CRT) for a patient with cancer treatment-induced heart failure?	a. Yes b. No c. Other, … d. Not applicable	a. 53 (58.9%) b. 8 (8.9%) c. 11 (12.2%) d. 18 (20.0%)

The online survey was created at the platform ‘KI survey’, designed and maintained by the Karolinska Institute, Stockholm, Sweden. This platform is an independent service; data are stored on a protected database and are not used for commercial purposes. Email addresses are entered in the platform to distribute the invitations to the selected candidates. The email addresses are not connected to the completed surveys, enabling anonymity of the survey responders.

The survey was sent out November 2nd 2018, and two reminders were sent to the non-responders (November 17th and November 28th).

## Results

### Demographics

**Fig. 1:**
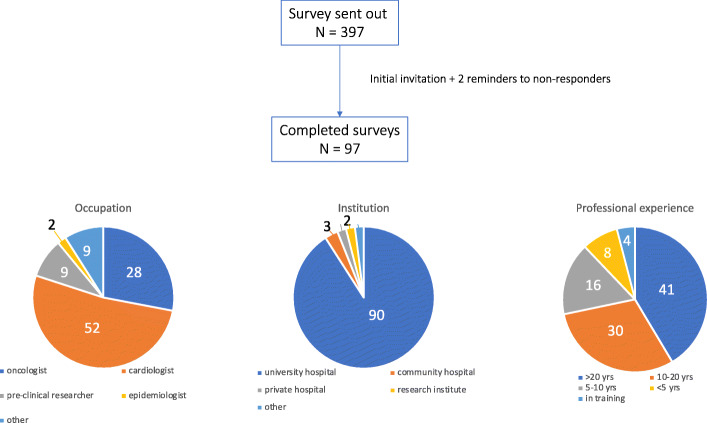
Accrual of participants and basic demographics

**Fig. 2:**
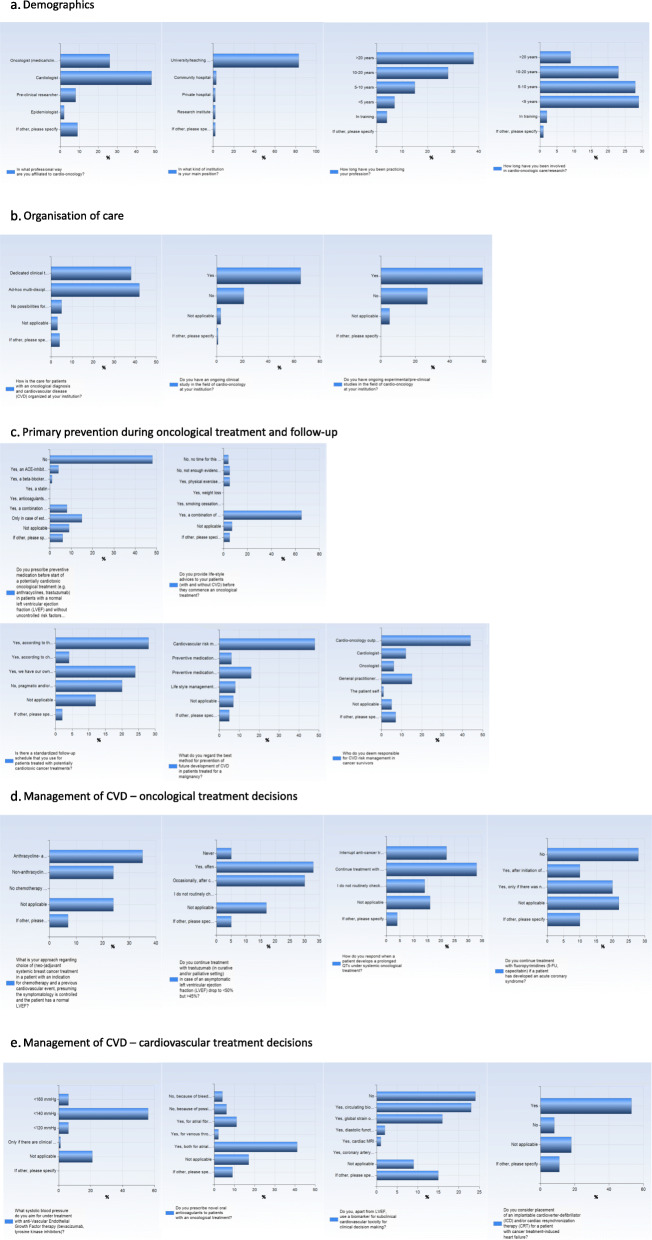
Overview of answers to questions in online cross-sectional survey among cardio-oncology experts. All questions and possible multiple-choice answers are presented in Table 1. For each question, only one of the answers could be selected. As an alternative, there was the possibility to write free text instead of choosing one of the available options for every multiple-choice question

Ninety-three respondents (25%) of the 372 experts approached, completed in the online questionnaire (Fig. 1). Of these 93 respondents, most were clinicians (Fig. 1, Table 1). The remaining 9% had positions such as nurse-researchers, pharmacologist, clinical and psychological researchers. Figure 2 provides an overview of the answers, and Additional file 1 provides the exact number of responses in the multiple-choice questions and the responses given in the open text fields.

Ninety percent of the participants worked at a university/teaching hospital, and most had over 10 years of clinical experience whereas 4% still were in training. About one third had over 10 years of experience in cardio-oncologic care and/or research. Little over half (of the respondents were male.

### Organization of care

Most of the respondents had access to multidisciplinary conferences at their institution; either as scheduled meetings (41%) or ad-hoc discussions and referrals (46%), but a small percentage (5%) mentioned lack of availability for multidisciplinary interaction.

The majority of respondents had ongoing clinical trials in the field of cardio-oncology at their clinic. Moreover, little over half had relevant pre-clinical and/or experimental studies that were currently ongoing.

### Primary prevention during oncological treatment

#### Preventive medication before and during oncologic treatment

The question asked to the experts was ‘Do you prescribe preventive medication before start of a potentially cardiotoxic oncological treatment (e.g. anthracyclines, trastuzumab) in patients with a normal left ventricular ejection fraction (LVEF) and without uncontrolled risk factors for CVD?

About half of the respondents do not routinely prescribe preventive medication before start of a potentially cardiotoxic oncological treatment in patients with a normal LVEF without uncontrolled risk factors for CVD. One in ten routinely initiate treatment with either an ACEi or angiotensin receptor antagonist (ARB) or a combination of ACEi or ARB with beta-blocker/statin and/or anticoagulants and 17% prescribe preventive medication only in case of an estimated increased risk for development of cardiotoxicity, based on published risk estimation scores [12, 13]. Some respondents mentioned a cardiologist should decide in this matter, and one respondent mentioned he/she applies the coronary artery calcification score (CAC) for decision-making.

#### Life-style interventions before and/or during treatment initiation

Almost three-quarter of respondents report providing recommendations on healthy lifestyle, such as physical exercise, weight loss and smoking cessation. On the contrary, 5% reportedly refrain from such recommendations due to lack of robust scientific evidence to support such interventions, and equal number does not give such recommendations due to time limitations. Some respondents report to offer exercise counseling to their patients.

### Primary prevention during follow-up

#### Strategies for prevention of CVD after cancer treatment completion

More than half of respondents (60%) have a routine follow-up schedule for patients treated with potentially cardiotoxic treatments. The majority uses the ASCO guidelines on prevention of cardiac dysfunction in cancer survivors, alternatively constructed local/regional guidelines.

Interestingly, almost half of the respondents consider a cardio-oncology outpatient clinic the appropriate forum for cardiovascular risk management (CVRM) in cancer survivors. Some respondents indicate that this should be primarily performed by a general practitioner, and few mention that this is a joint effort between general practitioners and cardiologists. Only one respondent deems the patient itself should be responsible for CVRM.

About half of respondents regard cardiovascular risk factor management according to general CVD guidelines the best prevention strategy of future development of CVD in patients treated for a malignancy. Almost a quarter sees a role for preventive medication, such as ACEi, ARB or beta-blockade in prevention of future CVD, whereas one fifth of all respondents would prescribe such medications only to patients with increase in troponins and/or NT-proBNP. Moreover, some support a primary role in life-style modifications such as weight loss, increased physical exercise and smoking cessation.

### Management of CVD – oncological treatment decisions

Table 2 provides an overview of the answers to the following eight questions subdivided by respondents’ occupation, i.e. oncologists vs. non-oncologists and cardiologists vs. non-cardiologists.

**Table 2:** Answers on questions covering specific oncological and cardiological treatment decisions, subdivided by responders’ profession and compared to the other respondents (i.e., all non-oncologists and non-cardiologists)

		Oncologists (*N* = 26)	Other respondents (*N* = 67)
What is your approach regarding choice of (neo-)adjuvant systemic breast cancer treatment in a patient with an indication for chemotherapy and a previous cardiovascular event, presuming the symptomatology is controlled and the patient has a normal LVEF?	a. Anthracycline- and taxane-based chemotherapy b. Non-anthracycline containing chemotherapy c. No chemotherapy because of co-morbidity d. Other, … e. Not applicable	a. 10 (38.5%) b. 12 (46.2%) c. 0 d. 0 e. 4 (15.4%)	a. 25 (39.1%) b. 12 (18.8%) c. 0 d. 7 (10.9%) e. 20 (31.3%)
Do you continue treatment with trastuzumab (in curative and/or palliative setting) in case of an asymptomatic left ventricular ejection fraction (LVEF) drop to < 50% but **≥**45%?	a. Never b. Yes, often c. Occasionally, after consultation with cardiologist when adequate anti-congestive treatment is initiated d. I do not routinely check LVEF during trastuzumab treatment if patients do not have any clinical signs of heart failure e. Other, … f. Not applicable	a. 1 (3.8%) b. 4 (15.4%) c. 16 (61.5%) d. 0 e. 0 f. 4 (15.4%)	a. 4 (6.3%) b. 29 (45.3%) c. 14 (21.9%) d. 0 e. 4 (6.3%) f. 13 (20.3%)
How do you respond when a patient develops a prolonged QTc under systemic oncological treatment?	a. Interrupt anti-cancer treatment, follow-up QTc and re-initiate only after QTc has normalized b. Continue treatment with continued monitoring of QTc c. I do not routinely check QTc under anti-cancer treatment d. Other, … e. Not applicable	a. 11 (42.3%) b. 5 (19.2%) c. 8 (30.8%) d. 0 e. 2 (7.7%)	a. 11 (17.5%) b. 28 (44.4%) c. 6 (9.5%) d. 4 (6.3%) e. 14 (22.2%)
Do you continue treatment with fluoropyrimidines (5-FU, capecitabin) if a patient has developed an acute coronary syndrome?	a. No b. Yes, after initiation of a calcium-antagonist c. Yes, only if there was no enzymatic myocardial infarction and if anti-angina treatment is started d. Other, … e. Not applicable	a. 11 (42.3%) b. 1 (3.8%) c. 7 (26.9%) d. 2 (7.7%) e. 5 (19.2%)	a. 17 (27.0%) b. 9 (14.3%) c. 13 (20.6%) d. 8 (12.7%) e. 17 (27.0%)
		**Cardiologists (*N*** **= 48)**	**Other respondents (*N*** **= 45)**
What systolic blood pressure do you aim for under treatment with anti-Vascular Endothelial Growth Factor therapy (bevacizumab, tyrosine kinase inhibitors)?	a. < 160 mmHg b. < 140 mmHg c. < 120 mmHg d. Only if there are clinical symptoms of hypertension and/or proteinuria e. Other, … f. Not applicable	a. 2 (4.2%) b. 41 (85.4%) c. 4 (8.3%) d. 0 e. 0 f. 4 (4.2%)	a. 4 (9.3%) b. 18 (41.9%) c. 2 (4.7%) d. 1 (2.3%) e. 0 f. 18 (41.9%)
Do you prescribe novel oral anticoagulants to patients with an oncological treatment?	a. No, because of bleeding risk b. No, because of possible interactions with oncological treatment c. Yes, for atrial fibrillation d. Yes, for venous thrombo-embolism e. Yes, both for atrial fibrillation and venous thrombo-embolism f. Other, … g. Not applicable	a. 2 (4.2%) b. 9 (18.8%) c. 0 d. 0 e. 28 (58.3%) f. 6 (12.5%) g. 3 (6.3%)	a. 4 (9.3%) b. 4 (9.3%) c. 2 (4.7%) d. 2 (4.7%) e. 13 (30.2%) f. 3 (7.0%) g. 15 (34.9%)
Do you, apart from LVEF, use a biomarker for subclinical cardiovascular toxicity for clinical decision making?	a. No b. Yes, circulating biomarkers (NT-proBNP, troponins) c. Yes, global strain on echocardiography d. Yes, diastolic function on echocardiography (E/A-ratio, tissue velocities) e. Yes, cardiac MRI f. Yes, coronary artery calcification scores on computed tomography g. Other, … h. Not applicable	a. 9 (18.8%) b. 13 (27.1%) c. 10 (20.8%) d. 0 e. 1 (2.1%) f. 0 g. 14 (29.2%) – all combo’s h. 1 (2.1%)	a. 15 (34.9%) b. 10 (23.3%) c. 6 (14.0%) d. 2 (4.7%) e. 0 f. 0 g. 9 (20.9%) h. 0
Do you consider placement of an implantable cardioverter-defibrillator (ICD) and/or cardiac resynchronization therapy (CRT) for a patient with cancer treatment-induced heart failure?	a. Yes b. No c. Other, … d. Not applicable	a. 36 (75%) b. 3 (6.3%) c. 8 (16.7%) d. 1 (2.1%)	a. 17 (39.5%) b. 5 (11.6%) c. 17 (39.5%) d. 4 (9.3%)

#### (neo-) adjuvant systemic therapy for breast cancer

Little less than half of respondents recommend combination chemotherapy with anthracyclines and taxanes for a patient with a previous cardiovascular event, with controlled symptomatology and a normal LVEF, whereas about a quarter would recommend non-anthracycline containing chemotherapy. Oncologists tended to somewhat favor non-anthracycline based chemotherapy schemes compared to the rest of the respondents (Table 2).

#### Asymptomatic LVEF declines during trastuzumab treatment

In patients treated with trastuzumab (in curative and/or palliative setting) who develop an asymptomatic left ventricular ejection fraction (LVEF) drop to < 50% but ≥45%, almost two third of respondents will continue trastuzumab treatment, were half of this group does so after consultation with a cardiologist. Only some report they never continue trastuzumab under those circumstances. The oncologists as a group respond quite similar compared to the rest of the respondents.

#### Prolonged QTc

About half of respondents continue treatment with continued monitoring of QTc in case a patient develops a prolonged QTc under systemic oncological treatment, whereas some responded they never check QTc during anti-cancer treatment. A quarter of respondents interrupts oncological treatment and re-initiates therapy after QTc has normalized. Some respondents nuance the answer by stating that their approach strongly depends on the length of the QTc, and one continues anti-cancer treatment but discontinues supportive medication in the first hand. Interestingly, one third of oncologists reports never to check QTc during anticancer treatments (Table 2).

#### Cardiac ischemia related to fluoropyrimidines

About one third of respondents will not continue treatment with fluoropyrimidines (5-FU, capecitabin) after a patient has developed an acute coronary syndrome (Table 1), whereas almost half of the oncologists would not do so (Table 2). One in ten respondents in the group as a whole re-initiates therapy after a patient has received treatment with a calcium-antagonist, and about a quarter would only do so in case there was no enzymatic myocardial infarction and adequate anti-ischemic treatment is initiated. For this question, a quarter of respondents selected the option ‘not applicable’. One respondent mentioned doing genetic testing (presumably for DPYD-deficiency [RA]), one reported having a re-challenge protocol and one would only restart in case of clean angiography.

### Management of CVD – cardiovascular treatment decisions

#### Blood pressure during anti-VEGF therapy

The majority of respondents aim for a systolic blood pressure < 140 mmHg in patients with anti-Vascular Endothelial Growth Factor therapy (i.e., bevacizumab, tyrosine kinase inhibitors); over 80% of cardiologists agrees on this (Table 2). Only one respondent indicated he/she would initiate treatment in case of clinical symptoms of hypertension and/or proteinuria.

#### Novel oral anticoagulants (NOAC) for patients on oncological treatments

Most respondents prescribe NOACs for atrial fibrillation and/or venous thrombo-embolisms, whereas some (12 and 2%) do so for the respective indications. About one tenth of respondents (11%) does not prescribe NOACs because of other possible interactions with oncological treatments or increased bleeding risk, whereas a number of respondents answered that the decision of treatment with NOAC is highly dependent on various factors such as indication, underlying malignancy and oncological treatment.

#### Biomarkers for subclinical cardiovascular toxicity

About one quarter of respondents uses LVEF only for clinical decision-making, whereas little over half reports using additional biomarkers for this purpose. The most frequently used biomarkers are the circulating biomarkers NT-proBNP and troponins, followed by global longitudinal strain on echocardiography. In addition, about 17% of respondents indicated combination of different biomarkers for subclinical cardiovascular damage for clinical decision-making, mainly the combination of circulating biomarkers and strain. Cardiologists seem to favor use of other biomarkers in addition to LVEF more than the other group of respondents (Table 2).

#### Implantable cardioverter-defibrillator (ICD) and/or cardiac resynchronization therapy (CRT) for cancer treatment-induced heart failure

Over half of all respondents (59%) consider placement of an ICD and/or CRT for patients with cancer treatment-induced heart failure, whereas 12% indicate they do so depending on the prognosis of the malignancy. Cardiologists are clearly more in favor of considering ICD compared to the other respondents, as is apparent from Table 2.

## Discussion

In this article we describe the results of a cross-sectional online survey among cardio-oncology experts. The aim of our project was to study clinical practices and expert approaches to several clinical cardio-oncological dilemmas regarding prediction, prevention and treatment of CVD in adult cancer patients, where definitive management recommendations are as of yet lacking.

By sending out this online questionnaire, we were able to obtain many highly valuable insights into clinical cardio-oncology practices from international experts. We conclude that it is feasible to perform such a survey, as has been done in other fields of medicine among health-care professionals [17–19]. The fast majority (80%) of respondents were physicians, most of them cardiologists (50% of all respondents), with a long period of professional expertise as three quarter had over 10 years of professional experience. This is expected based on the method we chose to select potential respondents, those being first and/or last author of papers published in the research area of cardio-oncology. We believe that this group of respondentshas a firm clinical and scientific basis to provide insights in the clinical dilemmas discussed in our survey.

One in six respondents mentioned to use baseline predictive models for future CVD to decide on prescription of medication as primary prevention before start of an oncological treatment. Reliable predictive biomarkers, that enable an estimation of the risk of future CVD before an oncological treatment is initiated, are highly needed. Such biomarkers enable selection of patients who are candidates for primary prevention, intensified life-style modifications and/or screening for subclinical CVD. As of now, some published studies have investigated such predictive models in breast cancer patient cohorts [12–14], but methodological limitations and especially lack of external validation in independent patient cohorts make it as of yet hard to implement such scores in routine clinical practice. The possibilities and limitations of such predictive models are also summarized in the previously mentioned guidelines [15–18].

The majority of respondents mention that they provide recommendations for optimization of life-style related risk factors before a cancer treatment is started, and around 10% of respondents sees a role in interventions targeting life-style modifications for prevention of future CVD after completion of oncological treatments. Such risk factors for CVD include obesity, smoking, hypertension and dyslipidemia. Presence of those risk factors, that can actually be seen as shared risk factors for the development of CVD and cancer, is associated with a higher risk of treatment-related cardiovascular toxicity [12, 19, 20].

The guidelines on management and prevention of cardiac dysfunction in cancer patients [15–18] underscore the importance of screening for, and educating cancer survivors about, lifestyle modifications such as physical exercise and dietary habits leading to prevention of weight gain. None of the guidelines mentions smoking cessation, but this might also exert positive benefits for cardiovascular health in cancer survivors as well as decrease future cancer risk. An EBCTCG meta-analysis indicated an increased risk for late radiotherapy-induced cardiac toxicity in smokers [21], supporting pro-active recommendations for smoking cessation.

At the moment, no definite conclusions can be made on to what extent interventions targeting physical exercise or weight loss during or after cancer treatments decrease CVD in cancer survivors. To the best of our knowledge, there are no studies with a physical exercise intervention that used cardiovascular outcomes as primary endpoint. Some exploratory analyses of the existing studies have shown beneficial outcomes in terms of cardiovascular outcomes, such as preserved cardiorespiratory fitness [22], lower insulin levels [23] and less treatment-related tachycardia [24]. Intervention trials aiming to ameliorate treatment-related cardiovascular toxicity by a physical exercise intervention are ongoing. A Canadian study (NCT03131024) investigates the role of caloric restriction and physical exercise in LVEF reserve 2–3 weeks and 1 year after adjuvant treatment completion as primary endpoint. In addition, a French study (NCT02433067) has been initiated in patients undergoing systemic adjuvant treatment for early HER2-positive breast cancer, where patients are randomized between physical activity or standard of care; change in LVEF from baseline to 6 months after treatment start is primary endpoint of this trial.

Over half of respondents use, in addition to LVEF, (combinations of) additional biomarkers for subclinical cardiovascular toxicity in their clinical decision-making. The most used ones are circulating biomarkers, NT-proBNP and troponins, and global longitudinal strain on echocardiography. In addition, one in five of respondents added in the open text response option that they used combinations of those – in some cases combined with cardiac magnetic resonance imaging. The ESMO guideline discusses the role of the different biomarkers, and concludes that routine use of cardiac biomarkers (cardiac troponins, BNP and NT pro-BNP) for patients undergoing potentially cardiotoxic chemotherapy is not well established [18]. In addition, the guideline mentions the potential usefulness of strain on echocardiography, but no statements are made as to whether the use of such parameters can already be implemented in the clinic. Ideally, a combination score of (temporary) changes in biomarkers for subclinical cardiovascular damage could potentially serve as a tool to identify patients at increased risk for future CVD. To the best of our knowledge, studies investigating such biomarker combinations have as of yet not been initiated.

An important finding in our survey is the fact that there is quite some heterogeneity in management and treatment decisions regarding the clinical dilemmas discussed in the question, e.g. the routine use of biomarkers, management of fluoropyrimidine-vasospasm and primary prevention strategies. This heterogeneity could result in differences in standard of care, depending on the site where a patient is treated, which is an undesired situation and warrants the need for firm evidence supporting these clinical dilemmas.

The possibility of performing digital, web-based surveys where participants can participate in an anonymized setting has resulted in a widespread use of such instruments, in all kinds of different societal settings. In medicine, web-based questionnaires have been used to study common practices and physician’s attitudes towards a large number of different items. We selected potential respondents based on academic merits, and the responses in the survey revealed that the responders constituted a group of mainly physicians with a long clinical experience within cardio-oncology. Because of the nature of multiple-choice questions, being able to select only one answer that one regards most fitting, will there be a loss of nuance and information obtained from the survey. However, we tried to minimize this by providing possibilities for selecting a combined answering option, as well as an open text field option.

The applied method of selecting potential participants can be a source for bias as persons actively involved in cardio-oncology research may tend to favour a certain intervention that they have been studying. This could even be more pronounced as it is more likely that positive studies are submitted and accepted for publication. Additional sources for bias may originate from respondents being co-authors, coming from the same institution as well as presence of different of medical payer and provider systems that can put different constraints on practice.

A quarter of all invited respondentscompleted the survey after the initial invitation and two reminders. No financial or other incentives were provided, which might have resulted in higher response rates [25], and neither was an answering tracking system used, in line with findings that the use of this may negatively impact response rates [26]. Our response rate is somewhat lower than studies that have investigated response rates of physicians to web-based surveys, where percentages between 29 and 35% were found [25, 27]. Because of this response rate, we cannot exclude the possibility that nonresponse bias has influenced the current findings [28].

The questions selected for this survey have been composed by the clinical cardio-oncology team at our institution, with the aim to identify practices in clinical dilemmas in cardio-oncology. There may be suboptimal formulations and possibly, additional answers would have been even more appropriate to obtain maximal insight in this matter. For this reason, we provided open text options at each question. Individual responses to the respective questions are given in Additional file 1. The option ‘not applicable’ (N/A) was added to enable respondents to refrain from answering a question that was beyond their expertise. This might also have complicated the interpretation of the findings, because respondents unsure of the best option may also have made an ‘educated guess’ instead of selecting N/A.

The survey was specifically designed to be generic and not to focus, with the exception of one question on breast cancer therapy selection, on specific tumor types. Doing so may have impacted the response interpretation.

## Conclusions

In conclusion, a web-based survey was conducted among cardio-oncology experts to explore attitudes towards clinical dilemmas related to prediction, prevention and management of treatment-related cardiovascular toxicity in cancer patients, where high-quality evidence from the literature is still lacking. The answers provided in this survey have shed light on expert-based practices in clinical cardio-oncology dilemmas. Attitudes towards as well as discrepancies in those dilemmas are presented. Existing discrepancies clearly indicate the need for generation of high-quality data that allows for more evidence-based recommendations in the future.

## Supplementary information

**Additional file 1.**

## Data Availability

The datasets used and/or analysed during the current study are available in Additional file 1
