# Supplementary material for: Approaches to management of cardiovascular morbidity in adult cancer patients – cross-sectional survey among cardio-oncology experts
Source: Cardiooncology. 2020 Sep 1;6:16. doi: 10.1186/s40959-020-00070-y (PMC7460793; doi:10.1186/s40959-020-00070-y)

# Cardio-oncology Survey Karolinska

Respondents: 372
Answer Count: 93
Answer Frequency: 25.00 %

## In what professional way are you affiliated to cardio-oncology?

| In what professional way are you affiliated to cardio-oncology? | Number of Responses |
|---|---|
| Oncologist (medical/clinical/radiation therapy) | 26 (28.0%) |
| Cardiologist | 48 (51.6%) |
| Pre-clinical researcher | 8 (8.6%) |
| Epidemiologist | 2 (2.2%) |
| If other, please specify | 9 (9.7%) |
| Total | 93 (100.0%) |

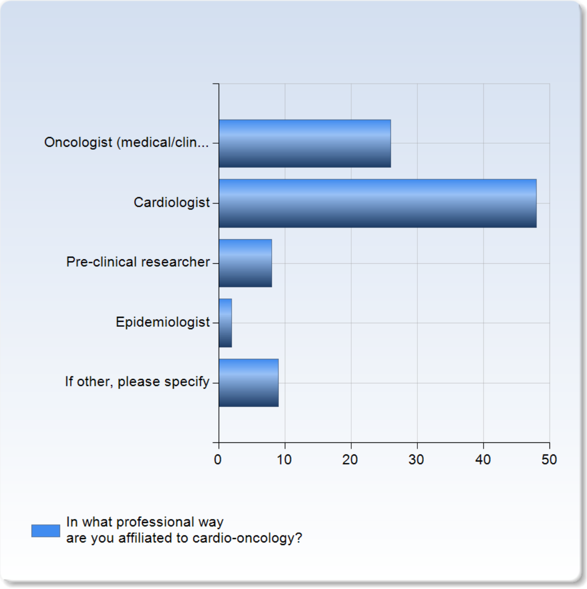


| | Mean | Standard Deviation | Coefficient of Variation | Min | Lower Quartile | Median | Upper Quartile | Max |
|---|---|---|---|---|---|---|---|---|
| In what professional way are you affiliated to cardio-oncology? | 2.1 | 1.1 | 53.6 % | 1.0 | 1.0 | 2.0 | 2.0 | 5.0 |

| If other, please specify |
|---|
| Paediatric oncologist |
| I did a clinical study assessing cardiac function during exercise |
| clinical researcher |
| angiologist |
| Academic researcher |
| Nurse Academic |
| Psychological researcher |
| Nurse Practitioner |
| Clinical Pharmacology |

## In what kind of institution is your main position?

| In what kind of institution is your main position? | Number of Responses |
|---|---|
| University/teaching hospital | 83 (90.2%) |
| Community hospital | 3 (3.3%) |
| Private hospital | 2 (2.2%) |
| Research institute | 2 (2.2%) |
| If other, please specify | 2 (2.2%) |
| Total | 92 (100.0%) |

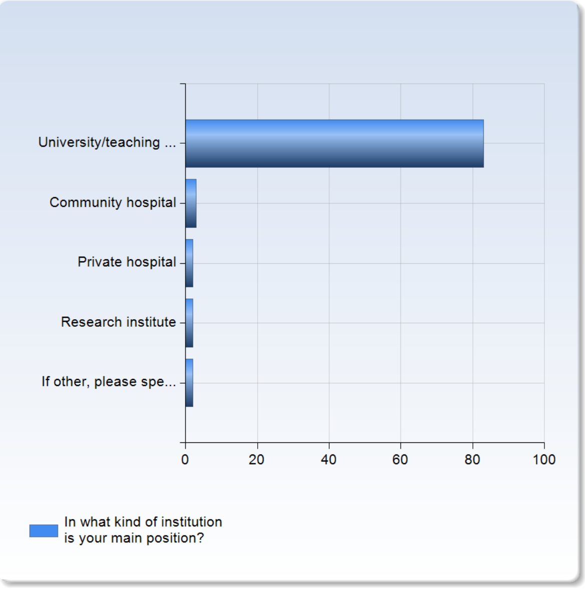


| | Mean | Standard Deviation | Coefficient of Variation | Min | Lower Quartile | Median | Upper Quartile | Max |
|---|---|---|---|---|---|---|---|---|
| In what kind of institution is your main position? | 1.2 | 0.8 | 64.0 % | 1.0 | 1.0 | 1.0 | 1.0 | 5.0 |

| If other, please specify |
|---|
| now retired - private practice |
| Community cardiology outpatient clinic |

Karolinska Institutet

## How long have you been practicing your profession?

| How long have you been practicing your profession? | Number of Responses |
|---|---|
| >20 years | 38 (41.3%) |
| 10-20 years | 28 (30.4%) |
| 5-10 years | 15 (16.3%) |
| <5 years | 7 (7.6%) |
| In training | 4 (4.3%) |
| If other, please specify | 0 (0.0%) |
| Total | 92 (100.0%) |

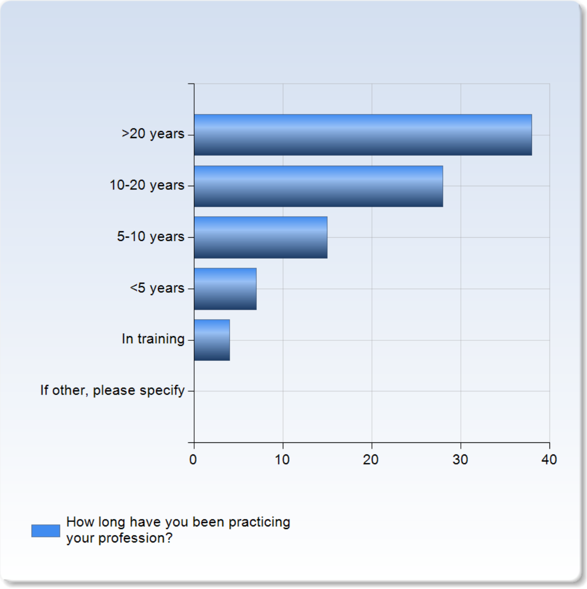


| | Mean | Standard Deviation | Coefficient of Variation | Min | Lower Quartile | Median | Upper Quartile | Max |
|---|---|---|---|---|---|---|---|---|
| How long have you been practicing your profession? | 2.0 | 1.1 | 55.8 % | 1.0 | 1.0 | 2.0 | 3.0 | 5.0 |

## How long have you been involved in cardio-oncologic care/research?

| How long have you been involved in cardio-oncologic care/research? | Number of Responses |
|---|---|
| >20 years | 9 (9.8%) |
| 10-20 years | 23 (25.0%) |
| 5-10 years | 28 (30.4%) |
| <5 years | 29 (31.5%) |
| In training | 2 (2.2%) |
| If other, please specify | 1 (1.1%) |
| Total | 92 (100.0%) |

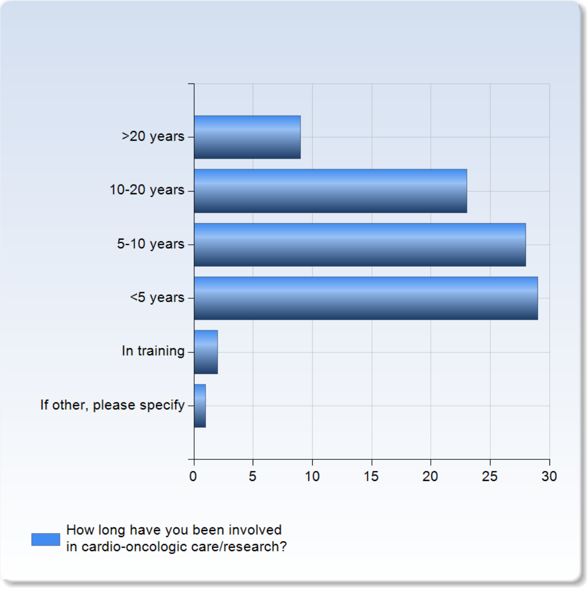


| | Mean | Standard Deviation | Coefficient of Variation | Min | Lower Quartile | Median | Upper Quartile | Max |
|---|---|---|---|---|---|---|---|---|
| How long have you been involved in cardio-oncologic care /research? | 2.9 | 1.1 | 36.4 % | 1.0 | 2.0 | 3.0 | 4.0 | 6.0 |

If other, please specify

Mentoring Madters student only

## What is your gender

| What is your gender | Number of Responses |
|---|---|
| Female | 34 (37.8%) |
| Male | 56 (62.2%) |
| Total | 90 (100.0%) |

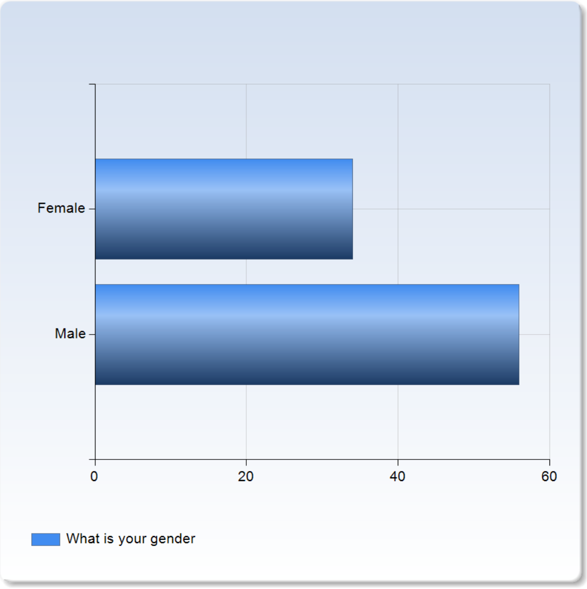


| | Mean | Standard Deviation | Coefficient of Variation | Min | Lower Quartile | Median | Upper Quartile | Max |
|---|---|---|---|---|---|---|---|---|
| What is your gender | 1.6 | 0.5 | 30.1 % | 1.0 | 1.0 | 2.0 | 2.0 | 2.0 |

# How is the care for patients with an oncological diagnosis and cardiovascular disease (CVD) organized at your institution?

| How is the care for patients with an oncological diagnosis and cardiovascular disease (CVD) organized at your institution? | Number of Responses |
|---|---|
| Dedicated clinical team with scheduled multi-disciplinary conferences | 38 (41.3%) |
| Ad-hoc multi-disciplinary discussions and referrals | 42 (45.7%) |
| No possibilities for this | 5 (5.4%) |
| Not applicable | 3 (3.3%) |
| If other, please specify | 4 (4.3%) |
| Total | 92 (100.0%) |

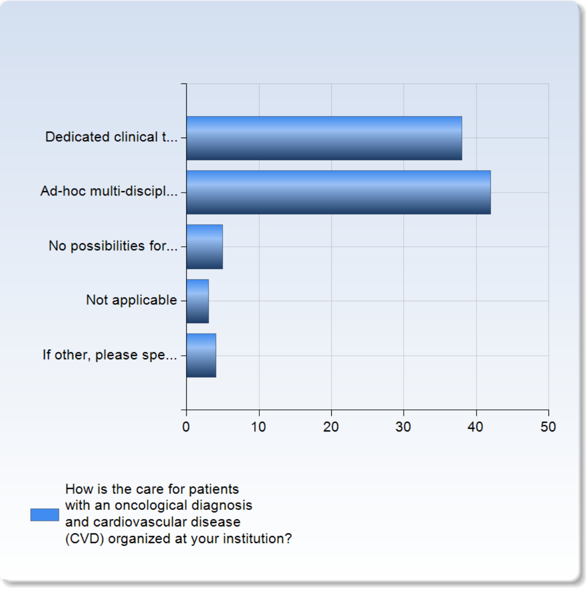


| | Mean | Standard Deviation | Coefficient of Variation | Min | Lower Quartile | Median | Upper Quartile | Max |
|---|---|---|---|---|---|---|---|---|
| How is the care for patients with an oncological diagnosis and cardiovascular disease (CVD) organized at your institution? | 1.8 | 1.0 | 53.7 % | 1.0 | 1.0 | 2.0 | 2.0 | 5.0 |

| If other, please specify |
|---|
| referral to dedicated cardiologists in Cardiology |
| Well taken care of up to 18 years of  age. After that we refer those in need of surveillance through our late effects clinic. |
| I think there is a specialty clinic but not sure given that oncologist has moved |
| Cerain providers specialize in this |

# Do you prescribe preventive medication before start of a potentially cardiotoxic oncological treatment (e.g. anthracyclines, trastuzumab) in patients with a normal left ventricular ejection fraction (LVEF) and without uncontrolled risk factors for cardiovascular disease (CVD)?

| Do you prescribe preventive medication before start of a potentially cardiotoxic oncological treatment (e.g. anthracyclines, trastuzumab) in patients with a normal left ventricular ejection fraction (LVEF) and without uncontrolled risk factors for cardiovascular disease (CVD)? | Number of Responses |
|---|---|
| No | 48 (52.7%) |
| Yes, an ACE-inhibitor/angiotensin receptor antagonist | 4 (4.4%) |
| Yes, a beta-blocker | 1 (1.1%) |
| Yes, a statin | 0 (0.0%) |
| Yes, anticoagulants | 0 (0.0%) |
| Yes, a combination of the abovementioned | 8 (8.8%) |
| Only in case of estimated increased risk for cardiotoxicity based on published risk scores (e.g., Ezaz et al, J Am Heart Assoc 2014; Herrmann et al, Mayo Clin Proc 2014) | 15 (16.5%) |
| Not applicable | 9 (9.9%) |
| If other, please specify | 6 (6.6%) |
| Total | 91 (100.0%) |

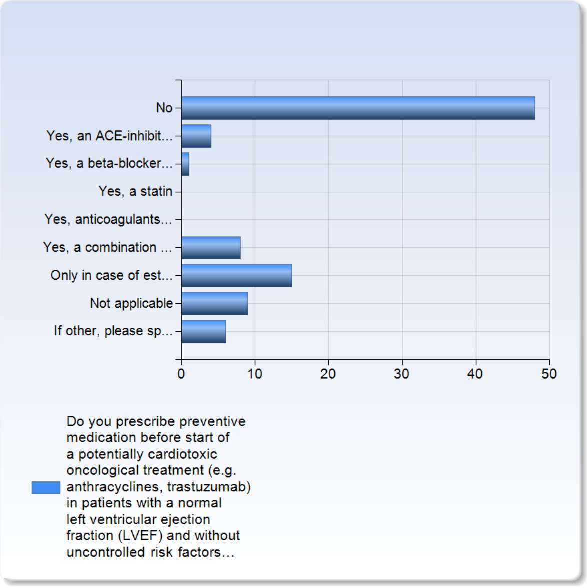


| | Mean | Standard Deviation | Coefficient of Variation | Min | Lower Quartile | Median | Upper Quartile | Max |
|---|---|---|---|---|---|---|---|---|
| Do you prescribe preventive medication before start of a potentially cardiotoxic oncological treatment (e.g. anthracyclines, trastuzumab) in patients with a normal left ventricular ejection fraction (LVEF) and without uncontrolled risk factors for cardiovascular disease (CVD)? | 4.2 | 3.8 | 90.7 % | 1.0 | 1.0 | 1.0 | 8.0 | 10.0 |

| If other, please specify |
|---|
| If I can, but the collaboration is still in childrens's shoes |
| If they are considered high risk, yes (prior anthra, Radiation, etc) and would first use a RAS inhibitor |
| Based on pre-CT clinical risk assessment |
| I refer to cardiologists who often do |
| I'm not a clinician, but I think that I should prescribe an ACE-inhibitor/angiotensin receptor antagonist |
| Do not know |

# Do you provide life-style advices to your patients (with and without CVD) before they commence an oncological treatment?

| Do you provide life-style advices to your patients (with and without CVD) before they commence an oncological treatment? | Number of Responses |
|---|---|
| No, no time for this | 4 (4.4%) |
| No, not enough evidence to support such recommendations | 5 (5.5%) |
| Yes, physical exercise | 5 (5.5%) |
| Yes, weight loss | 0 (0.0%) |
| Yes, smoking cessation | 0 (0.0%) |
| Yes, a combination of the abovementioned | 65 (71.4%) |
| Not applicable | 7 (7.7%) |
| If other, please specify | 5 (5.5%) |
| Total | 91 (100.0%) |

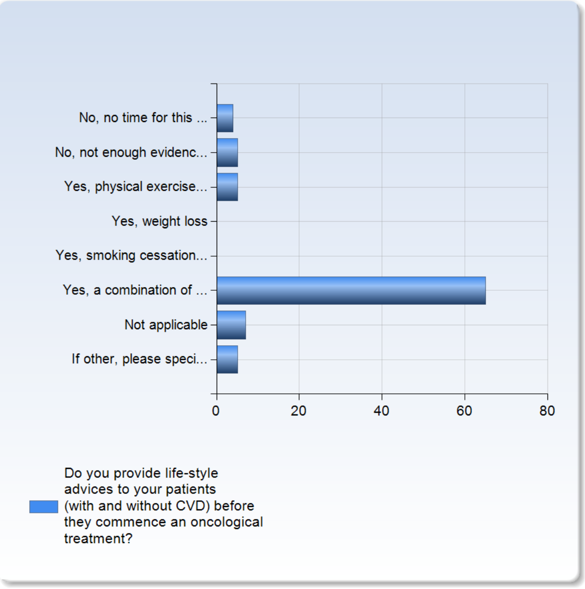


| | Mean | Standard Deviation | Coefficient of Variation | Min | Lower Quartile | Median | Upper Quartile | Max |
|---|---|---|---|---|---|---|---|---|
| Do you provide life-style advices to your patients (with and without CVD) before they commence an oncological treatment? | 5.6 | 1.6 | 29.0 % | 1.0 | 6.0 | 6.0 | 6.0 | 8.0 |

| If other, please specify |
|---|
| we are planning to do so |
| we are starting exercise counselling |
| I only see a subset of these patient in cardiology, but yes I do advise all of the above |
| Do not know |

Karolinska Institutet

# What is your approach regarding choice of (neo-)adjuvant systemic breast cancer treatment in a patient with an indication for chemotherapy and a previous cardiovascular event, presuming the symptomatology is controlled and the patient has a normal LVEF?

| What is your approach regarding choice of (neo-) adjuvant systemic breast cancer treatment in a patient with an indication for chemotherapy and a previous cardiovascular event, presuming the symptomatology is controlled and the patient has a normal LVEF? | Number of Responses |
|---|---|
| Anthracycline- and taxane-based chemotherapy | 35 (38.9%) |
| Non-anthracycline containing chemotherapy | 24 (26.7%) |
| No chemotherapy because of co-morbidity | 0 (0.0%) |
| Not applicable | 24 (26.7%) |
| If other, please specify | 7 (7.8%) |
| Total | 90 (100.0%) |

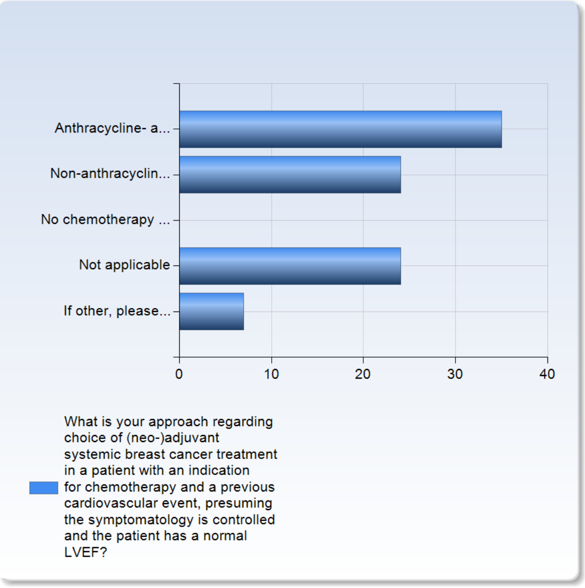


| | Mean | Standard Deviation | Coefficient of Variation | Min | Lower Quartile | Median | Upper Quartile | Max |
|---|---|---|---|---|---|---|---|---|
| What is your approach regarding choice of (neo-)adjuvant systemic breast cancer treatment in a patient with an indication for chemotherapy and a previous cardiovascular event, presuming the symptomatology is controlled and the patient has a normal LVEF? | 2.4 | 1.4 | 60.0 % | 1.0 | 1.0 | 2.0 | 4.0 | 5.0 |

| If other, please specify |
|---|
| I'm cardiologist |
| The oncologist decides |
| I am a cardiologist, so I advise about risk but dont dictate the chemo chosen.  I would suggest the best therapy for optimal cancer outcome |
| Anthracycline + trastuzumab |
| individualized |
| varies |
| Do not know |

# Do you continue treatment with trastuzumab (in curative and/or palliative setting) in case of an asymptomatic left ventricular ejection fraction (LVEF) drop to <50% but >45%?

| Do you continue treatment with trastuzumab (in curative and/or palliative setting) in case of an asymptomatic left ventricular ejection fraction (LVEF) drop to <50% but >45%? | Number of Responses |
|---|---|
| Never | 5 (5.6%) |
| Yes, often | 33 (36.7%) |
| Occasionally, after consultation with cardiologist when adequate anti-congestive treatment is initiated | 30 (33.3%) |
| I do not routinely check LVEF during trastuzumab treatment if patients do not have any clinical signs of heart failure | 0 (0.0%) |
| Not applicable | 17 (18.9%) |
| If other, please specify | 5 (5.6%) |
| Total | 90 (100.0%) |

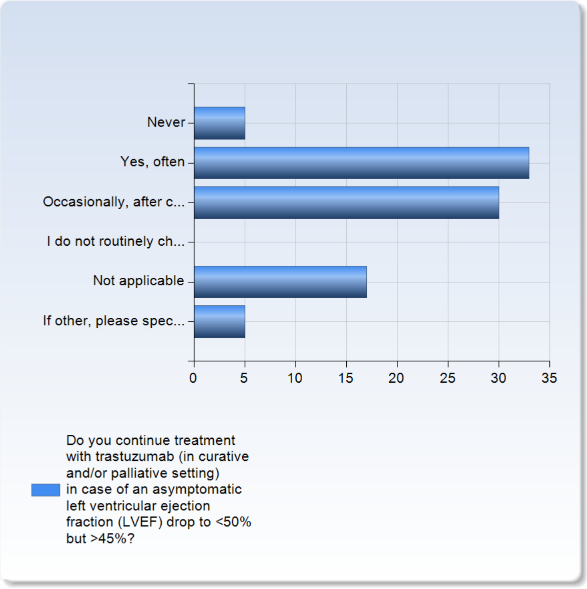


| | Mean | Standard Deviation | Coefficient of Variation | Min | Lower Quartile | Median | Upper Quartile | Max |
|---|---|---|---|---|---|---|---|---|
| Do you continue treatment with trastuzumab (in curative and/or palliative setting) in case of an asymptomatic left ventricular ejection fraction (LVEF) drop to <50% but >45%? | 3.1 | 1.4 | 44.5 % | 1.0 | 2.0 | 3.0 | 3.0 | 6.0 |

| If other, please specify |
|---|
| yes, but providing ARB at the same time |
| I advocate for continuing but it depends on oncologists comfort |
| I'm counseling the oncologist for this possibility |
| sometimes, with close monitoring |
| Stop if drop is more than 10% but restart if LVEF goes back to normal |

# How do you respond when a patient develops a prolonged QTc under systemic oncological treatment?

| How do you respond when a patient develops a prolonged QTc under systemic oncological treatment? | Number of Responses |
|---|---|
| Interrupt anti-cancer treatment, follow-up QTc and re-initiate only after QTc has normalized | 22 (24.7%) |
| Continue treatment with continued monitoring of QTc | 33 (37.1%) |
| I do not routinely check QTc under anti-cancer treatment | 14 (15.7%) |
| Not applicable | 16 (18.0%) |
| If other, please specify | 4 (4.5%) |
| Total | 89 (100.0%) |

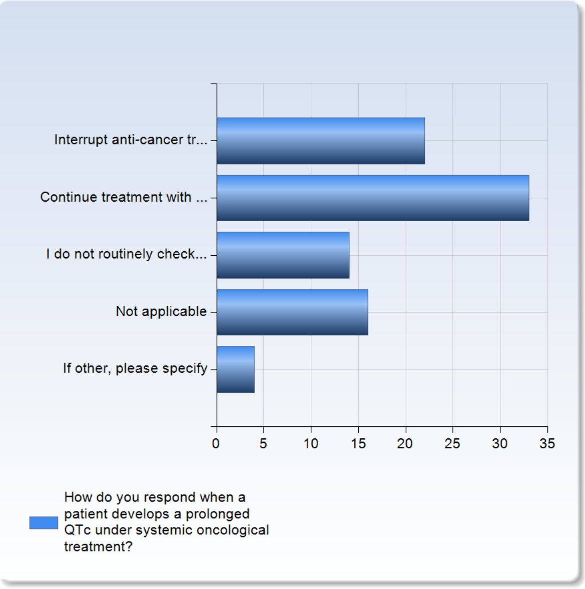


| | Mean | Standard Deviation | Coefficient of Variation | Min | Lower Quartile | Median | Upper Quartile | Max |
|---|---|---|---|---|---|---|---|---|
| How do you respond when a patient develops a prolonged QTc under systemic oncological treatment? | 2.4 | 1.2 | 48.9 % | 1.0 | 2.0 | 2.0 | 3.0 | 5.0 |

| If other, please specify |
|---|
| Depends on the QT |
| case by case basis. depends how long the QT is etc... |
| contine and stop offending supportive agents |
| Depends on degree of QTc prolongation and use of other QT-prolonging medications |

Karolinska Institutet

# Do you continue treatment with fluoropyrimidines (5-FU, capecitabin) if a patient has developed an acute coronary syndrome?

| Do you continue treatment with fluoropyrimidines (5-FU, capecitabin) if a patient has developed an acute coronary syndrome? | Number of Responses |
|---|---|
| No | 28 (31.1%) |
| Yes, after initiation of a calcium-antagonist | 10 (11.1%) |
| Yes, only if there was no enzymatic myocardial infarction and if anti-angina treatment is started | 20 (22.2%) |
| Not applicable | 22 (24.4%) |
| If other, please specify | 10 (11.1%) |
| Total | 90 (100.0%) |

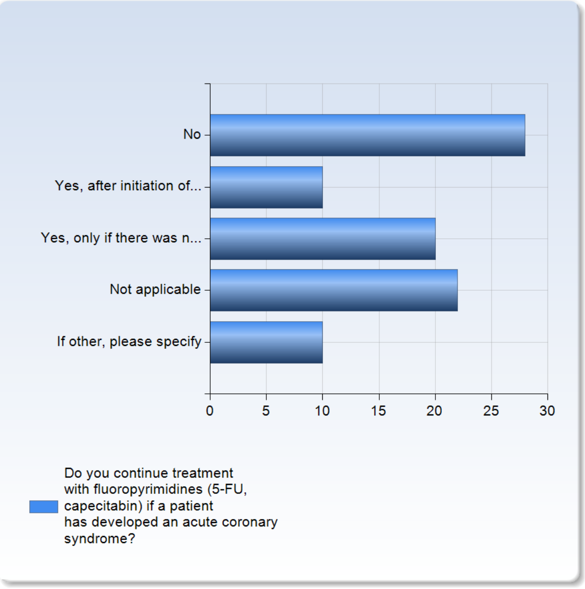


| | Mean | Standard Deviation | Coefficient of Variation | Min | Lower Quartile | Median | Upper Quartile | Max |
|---|---|---|---|---|---|---|---|---|
| Do you continue treatment with fluoropyrimidines (5-FU, capecitabin) if a patient has developed an acute coronary syndrome? | 2.7 | 1.4 | 51.7 % | 1.0 | 1.0 | 3.0 | 4.0 | 5.0 |

| If other, please specify |
|---|
| Do genetic testing, if negative will start anti-spastic treatment (i.e. amlodipin, nitrates). If symtoms again, stopp 5FU /analogues |
| I do a re-attempt after temporary discontinuation |
| individualized |
| We have rechallenge protocol |
| if patient revascularized |
| I would consider it after liaising with a cardiologist |
| sometimes |
| It depends on the coronary angiography |
| we consult a cardiologist |
| Yes, but only under CCB and nitrates with monitoring, which may require inpatient setup depending on severity of ACS |

# What systolic blood pressure do you aim for under treatment with anti-Vascular Endothelial Growth Factor therapy (bevacizumab, tyrosine kinase inhibitors)?

| What systolic blood pressure do you aim for under treatment with anti-Vascular Endothelial Growth Factor therapy (bevacizumab, tyrosine kinase inhibitors)? | Number of Responses |
|---|---|
| <160 mmHg | 6 (6.7%) |
| <140 mmHg | 56 (62.2%) |
| <120 mmHg | 6 (6.7%) |
| Only if there are clinical symptoms of hypertension and /or proteinuria | 1 (1.1%) |
| Not applicable | 21 (23.3%) |
| If other, please specify | 0 (0.0%) |
| Total | 90 (100.0%) |

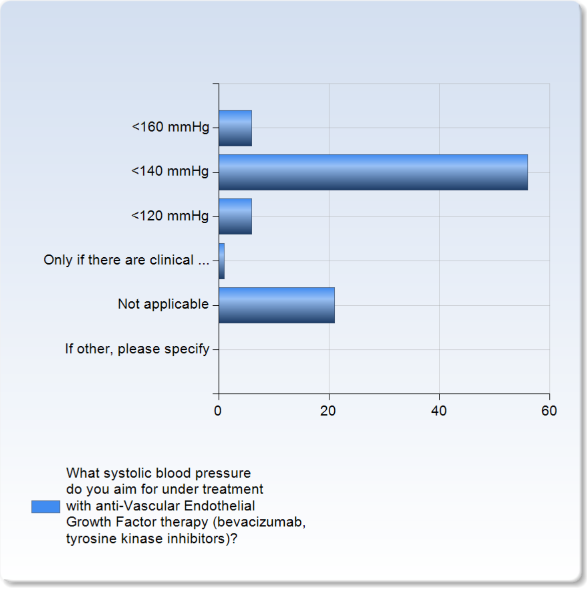


| | Mean | Standard Deviation | Coefficient of Variation | Min | Lower Quartile | Median | Upper Quartile | Max |
|---|---|---|---|---|---|---|---|---|
| What systolic blood pressure do you aim for under treatment with anti-Vascular Endothelial Growth Factor therapy (bevacizumab, tyrosine kinase inhibitors)? | 2.7 | 1.3 | 49.0 % | 1.0 | 2.0 | 2.0 | 3.0 | 5.0 |

# Do you prescribe novel oral anticoagulants to patients with an oncological treatment?

| Do you prescribe novel oral anticoagulants to patients with an oncological treatment? | Number of Responses |
|---|---|
| No, because of bleeding risk | 4 (4.4%) |
| No, because of possible interactions with oncological treatment | 6 (6.7%) |
| Yes, for atrial fibrillation | 11 (12.2%) |
| Yes, for venous thrombo-embolism | 2 (2.2%) |
| Yes, both for atrial fibrillation and venous thrombo-embolism | 41 (45.6%) |
| Not applicable | 17 (18.9%) |
| If other, please specify | 9 (10.0%) |
| Total | 90 (100.0%) |

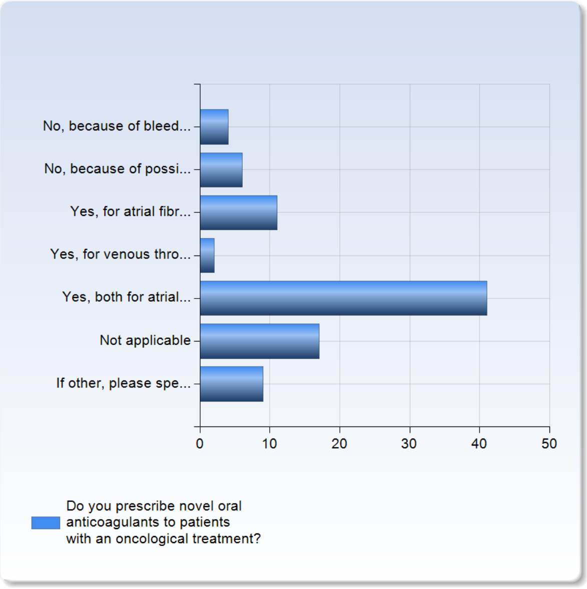

| | Mean | Standard Deviation | Coefficient of Variation | Min | Lower Quartile | Median | Upper Quartile | Max |
|---|---|---|---|---|---|---|---|---|
| Do you prescribe novel oral anticoagulants to patients with an oncological treatment? | 4.7 | 1.5 | 32.5 % | 1.0 | 4.5 | 5.0 | 6.0 | 7.0 |

| If other, please specify |
|---|
| depends on indication and type of cancer |
| No, LMWH or VKA. If indicated (AF, VT) |
| Not used in the paediatric setting |
| this is a poor question |
| Depnds on the case |
| Send to a VTE clinic |
| after eview of drug drug interactions |
| sometimes AF |
| it depends on other comorbidities especially with atrial fibrillation |

## Do you, apart from LVEF, use a biomarker for subclinical cardiovascular toxicity for clinical decision making?

| Do you, apart from LVEF, use a biomarker for subclinical cardiovascular toxicity for clinical decision making? | Number of Responses |
|---|---|
| No | 24 (26.7%) |
| Yes, circulating biomarkers (NT-proBNP, troponins) | 23 (25.6%) |
| Yes, global strain on echocardiography | 16 (17.8%) |
| Yes, diastolic function parameters on echocardiography (E/A-ratio, tissue velocities) | 2 (2.2%) |
| Yes, cardiac MRI | 1 (1.1%) |
| Yes, coronary artery calcification scores on computed tomography | 0 (0.0%) |
| Not applicable | 9 (10.0%) |
| If other, please specify | 15 (16.7%) |
| Total | 90 (100.0%) |

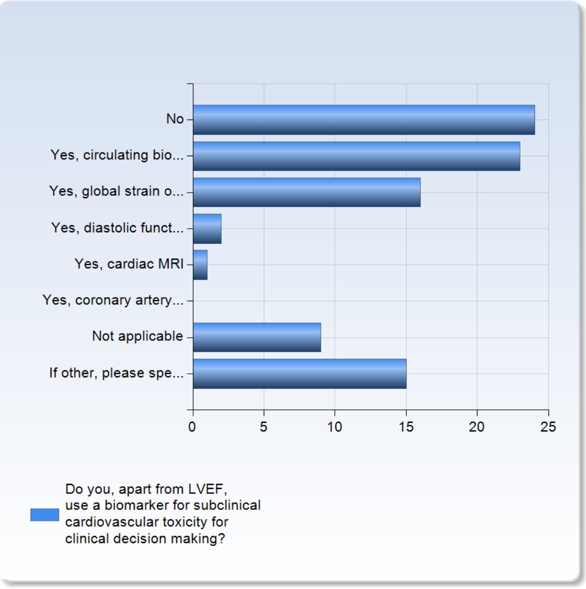


| | Mean | Standard Deviation | Coefficient of Variation | Min | Lower Quartile | Median | Upper Quartile | Max |
|---|---|---|---|---|---|---|---|---|
| Do you, apart from LVEF, use a biomarker for subclinical cardiovascular toxicity for clinical decision making? | 3.5 | 2.6 | 75.8 % | 1.0 | 1.0 | 2.0 | 7.0 | 8.0 |

| If other, please specify |
|---|
| not yet |
| echocardiography parameters |
| A combination of above depending on what insurance company will permit for testing; can be difficult to get MRI approvals |
| I use troponins, BNP, strain, CT calcium score and occasionally MRI |
| Yes, strain, troponin and occationally MRI |
| All of them depending on scenario |
| Combination of above |
| 2-4 |
| Combination of strain, diastolic parameters, and NTproBNP |
| Combination of above |
| yes, many biomarkers |
| NT-proBNP + Trop + strain |
| Combination of biomarkers and strain |
| Strain and troponins |
| Biomarkers AND GLS |

## What do you regard the best method for prevention of future development of CVD in patients treated for a malignancy?

| What do you regard the best method for prevention of future development of CVD in patients treated for a malignancy? | Number of Responses |
|---|---|
| Cardiovascular risk management according to general guidelines, with treatment based on accepted thresholds (blood pressure, lipid profile, glucose) | 48 (53.3%) |
| Preventive medication such as ACE-inhibitors, ARBs, beta-blockade independent of risk factors | 6 (6.7%) |
| Preventive medication such as ACE-inhibitors, ARBs, beta-blockade in patients with rises in troponin /NT-proBNP | 16 (17.8%) |
| Life style management (e.g., physical exercise, weight loss) | 8 (8.9%) |
| Not applicable | 7 (7.8%) |
| If other, please specify | 5 (5.6%) |
| Total | 90 (100.0%) |

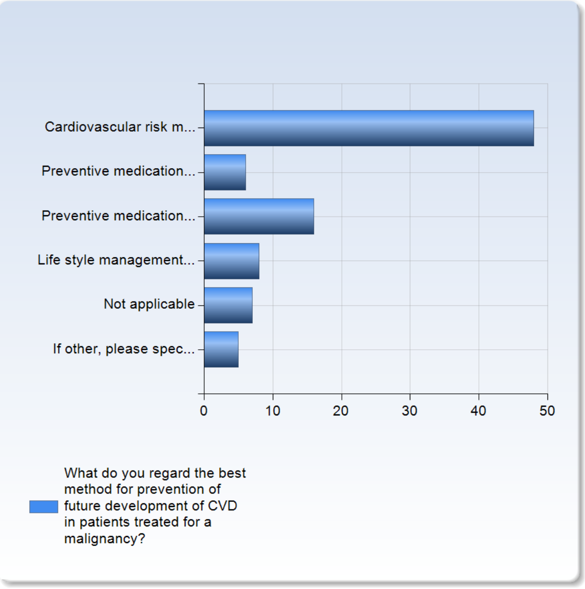


| | Mean | Standard Deviation | Coefficient of Variation | Min | Lower Quartile | Median | Upper Quartile | Max |
|---|---|---|---|---|---|---|---|---|
| What do you regard the best method for prevention of future development of CVD in patients treated for a malignancy? | 2.3 | 1.6 | 70.9 % | 1.0 | 1.0 | 1.0 | 3.0 | 6.0 |

| If other, please specify |
| --- |
| Fibrosis on Cardiac MRI |
| Combination of the above |
| I would do optimal risk management according to guidelines but specific high risk characteristics to cancer therapy, I would use preventive strategies |
| Life-style management plus CV risk management according GLs |
| Cardiovascular risk management according to general guidelines, with treatment initiation based on accepted thresholds (blood pressure, lipid profile, glucose); Preventive medication such as ACE-inhibitors, ARBs, beta-blockade in patients with rises in troponin/NT-proBNP; Life style management (e.g., physical exercise, weight loss) |

## Do you consider placement of an implantable cardioverter-defibrillator (ICD) and /or cardiac resynchronization therapy (CRT) for a patient with cancer treatment-induced heart failure?

| Do you consider placement of an implantable cardioverter-defibrillator (ICD) and/or cardiac resynchronization therapy (CRT) for a patient with cancer treatment-induced heart failure? | Number of Responses |
| --- | --- |
| Yes | 53 (58.9%) |
| No | 8 (8.9%) |
| Not applicable | 18 (20.0%) |
| If other, please specify | 11 (12.2%) |
| Total | 90 (100.0%) |

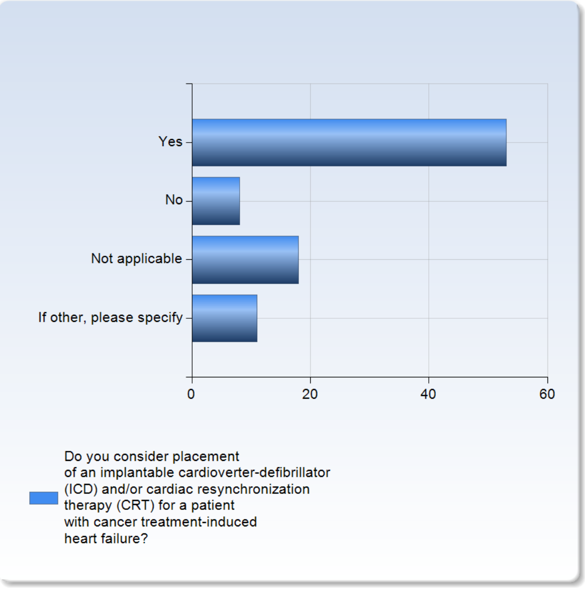


| | Mean | Standard Deviation | Coefficient of Variation | Min | Lower Quartile | Median | Upper Quartile | Max |
| --- | --- | --- | --- | --- | --- | --- | --- | --- |
| Do you consider placement of an implantable cardioverter-defibrillator (ICD) and /or cardiac resynchronization therapy (CRT) for a patient with cancer treatment-induced heart failure? | 1.9 | 1.1 | 60.8 % | 1.0 | 1.0 | 1.0 | 3.0 | 4.0 |

| If other, please specify |
|---|
| if the prognosis of cancer is good, otherwise no |
| depends life expectancy |
| No experience with this |
| depends |
| We routinely implant ICDs and even do heart transplant if the cancer is cured |
| yes according to cancer prognosis |
| it depends |
| A cardiologist would advise on this |
| Depends life expectency |
| it depends on other comorbidities |
| we willlet a cardiologist decide |

## Is there a standardized follow-up schedule that you use for patients treated with potentially cardiotoxic cancer treatments?

| Is there a standardized follow-up schedule that you use for patients treated with potentially cardiotoxic cancer treatments? | Number of Responses |
|---|---|
| Yes, according to the ASCO guideline (Prevention and Monitoring of Cardiac Dysfunction in Survivors of Adult Cancers, 2016) | 28 (31.1%) |
| Yes, according to childhood cancer survivorship guidelines | 4 (4.4%) |
| Yes, we have our own local/regional guideline | 24 (26.7%) |
| No, pragmatic and/or individualized schemes | 20 (22.2%) |
| Not applicable | 12 (13.3%) |
| If other, please specify | 2 (2.2%) |
| Total | 90 (100.0%) |

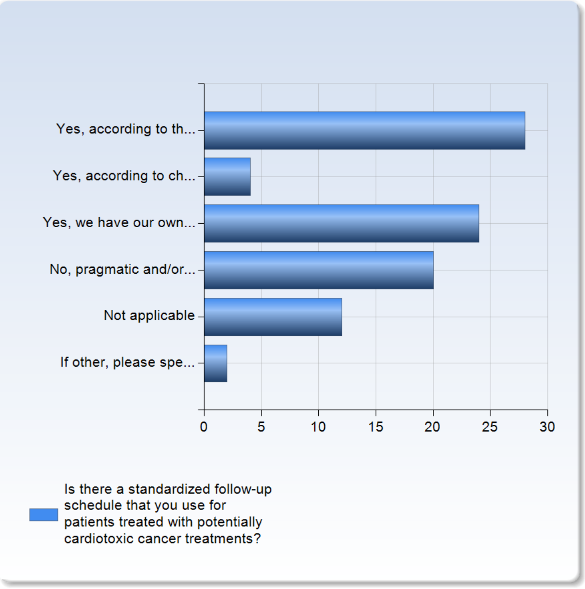


| | Mean | Standard Deviation | Coefficient of Variation | Min | Lower Quartile | Median | Upper Quartile | Max |
|---|---|---|---|---|---|---|---|---|
| Is there a standardized follow-up schedule that you use for patients treated with potentially cardiotoxic cancer treatments? | 2.9 | 1.5 | 52.0 % | 1.0 | 1.0 | 3.0 | 4.0 | 6.0 |

| If other, please specify |
|---|
| Shared decison making with the patient; often they may be overwhelmed by their cancer condition and are not excited about followup for the cardiovascular prevention |
| Both ASCO and local regional guidelines |

# Who do you deem responsible for CVD risk management in cancer survivors

| Who do you deem responsible for CVD risk management in cancer survivors | Number of Responses |
|---|---|
| Cardio-oncology outpatient clinic | 44 (48.9%) |
| Cardiologist | 12 (13.3%) |
| Oncologist | 6 (6.7%) |
| General practitioner | 15 (16.7%) |
| The patient self | 1 (1.1%) |
| Not applicable | 5 (5.6%) |
| If other, please specify | 7 (7.8%) |
| Total | 90 (100.0%) |

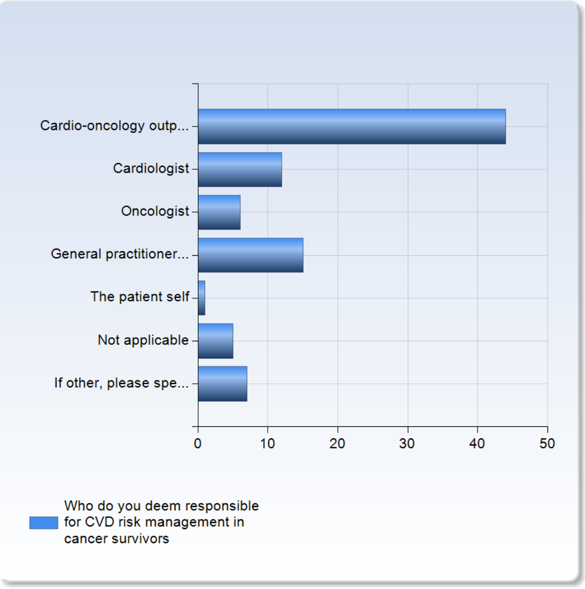


| | Mean | Standard Deviation | Coefficient of Variation | Min | Lower Quartile | Median | Upper Quartile | Max |
|---|---|---|---|---|---|---|---|---|
| Who do you deem responsible for CVD risk management in cancer survivors | 2.6 | 2.0 | 77.2 % | 1.0 | 1.0 | 2.0 | 4.0 | 7.0 |

| If other, please specify |
|---|
| A joint venture between the late effects clinic, our cardiologists and the patient |
| We have a cancer survivorship clinic |
| The collaboration between Oncologist and Cardiologist |
| Patient itself + GPs + General  Cardiologists + Cardio-oncology specialists |
| Oncologist-GP-Patient |
| All of the above; not all of the patients have access to a cardio-oncology clinic |
| Cardio-Oncology service and GP |

# Do you have an ongoing clinical study in the field of cardio-oncology at your institution?

| Do you have an ongoing clinical study in the field of cardio-oncology at your institution? | Number of Responses |
|---|---|
| Yes | 65 (72.2%) |
| No | 21 (23.3%) |
| Not applicable | 3 (3.3%) |
| If other, please specify | 1 (1.1%) |
| Total | 90 (100.0%) |

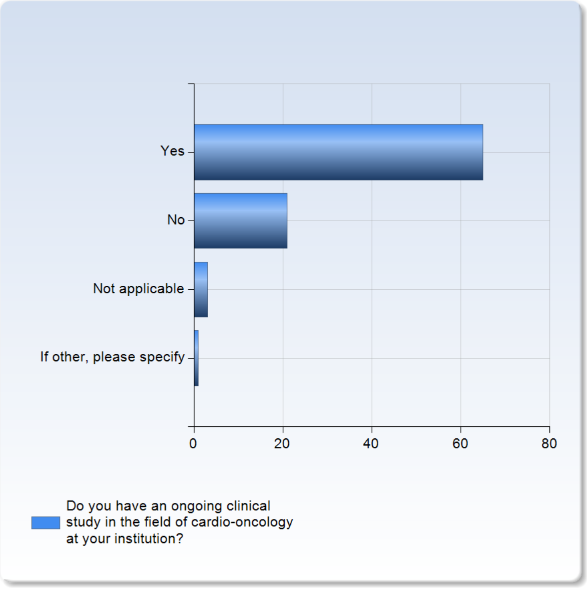


| | Mean | Standard Deviation | Coefficient of Variation | Min | Lower Quartile | Median | Upper Quartile | Max |
|---|---|---|---|---|---|---|---|---|
| Do you have an ongoing clinical study in the field of cardio-oncology at your institution? | 1.3 | 0.6 | 45.0 % | 1.0 | 1.0 | 1.0 | 2.0 | 4.0 |

| If other, please specify |
|---|
| PhD project studying current practice at my university |

# Do you have ongoing experimental/pre-clinical studies in the field of cardio-oncology at your institution?

| Do you have ongoing experimental/pre-clinical studies in the field of cardio-oncology at your institution? | Number of Responses |
|---|---|
| Yes | 59 (64.8%) |
| No | 27 (29.7%) |
| Not applicable | 5 (5.5%) |
| If other, please specify | 0 (0.0%) |
| Total | 91 (100.0%) |

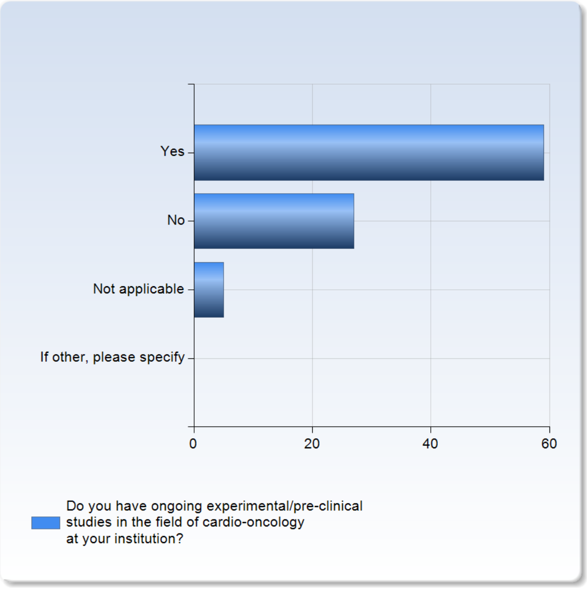

Supplement: Supplementary file 1 — Additional file 1. [file 40959_2020_70_MOESM1_ESM.pdf]
